# Retrieval of displaced implants inside the maxillary sinus: two case reports and a short review

**DOI:** 10.1186/s40729-019-0173-7

**Published:** 2019-06-04

**Authors:** Maria Gnigou, Lampros Goutzanis, Stavros Sarivalasis, Vasilios Petsinis

**Affiliations:** 1 Dental School Aristotle University of Thessaloniki, Kazantzaki 10A, 54628 Menemeni, Thessaloniki Greece; 2 Dental School National and Kapodistrian University of Athens, Zalogou 31, 18120 Korydallos, Athens Greece; 3 Dental School National and Kapodistrian University of Athens, Velestinou 9, Agios Dimitrios, 17343 Athens, Greece; 4 Dental School National and Kapodistrian University of Athens, Thermopylon 5, 16232 Vyronas, Athens Greece

## Abstract

**Objective:**

The aims of this paper are to demonstrate two cases of implant migration into the maxillary sinus and to give a short review of the literature on this subject.

**Clinical procedure:**

Two patients were diagnosed with implant migration into the maxillary sinus. After thorough radiographic examination which revealed the exact position of the implants inside the maxillary sinus, removal was performed through a bony window in the anterior-lateral aspect of the maxillary sinus for both cases.

**Discussion:**

Implant displacement into the maxillary sinus can occur intraoperatively or postoperatively either prior to implant loading or after functional loading. Several actors can lead to this complication differing according to the stage of the displacement.

Management of this complication is achieved using four surgical techniques: a. Functional endoscopic sinus surgery, b. intraoral removal by the Caldwell-Luc technique, c. removal through the alveolar bone, d. combination of the last two techniques. If implant displacement into the maxillary sinus remains untreated, it can lead to several complications with various effects.

**Conclusion:**

Migration of dental implants into the maxillary sinus is a rare but severe complication which must be treated as soon as possible.

## Introduction

Over the past three decades, implant-supported prosthesis has become very popular for the rehabilitation of edentulousness. However, alveolar bone resorption along with other anatomical changes that occur after tooth loss may cause difficulties concerning rehabilitation with the use of implants.

More specifically, implant rehabilitation of the posterior maxilla can be even more challenging due to poor bone quality (type IV bone [1]), high bone resorption, thin cortical bone [2], as well as extended pneumatization of the sinus [3].These factors pose certain difficulties during implant placement in the maxilla and can lead to several complications. One rare but severe complication is implant displacement into the paranasal sinuses. There are reports of implant migration into the sphenoid [4] or the ethmoid [5, 6] sinus, but most commonly displacement occurs into the maxillary sinus [1–3, 7–13].

Prominent causes of this complication are anatomical difficulties combined with surgical inexperience [7, 8]. Moreover, placement of dental implants without sinus lifting procedure in highly pneumatized sinuses, application of heavy force during implant insertion, the existence of untreated perforation of the antral base after completion of drilling sequence, as well as excessive tapping during sinus osteotomy are some of the mechanisms resulting in implant migration [7, 8]. On the other hand, implant perforation of the sinus floor mucosa no greater than 2 mm, causes spontaneous recovery of the sinus membrane and coverage of the dental implant, based on clinical and experimental studies [14, 15].

The present paper demonstrates two cases of dental implant displacement into the maxillary sinus, as well as surgical management of the cases and a short literature review on this subject.

## Cases report

### Case 1

A 73-year-old male with edentulous maxilla was referred to the University Department of Oral and Maxillofacial Surgery of the “Evaggelismοs” general hospital due to a migrated implant into the right maxillary sinus. The patient suffered from chronic obstructive pulmonary disease and therefore he had quitted smoking. Five years ago he underwent dental rehabilitation with the placement of six implants in the maxilla and four in the mandible. In less than 2 months after initial placement, all implants had failed to osseointegrate. A year and a half later the patient underwent guided bone regeneration with bovine-derived xenograft and 7 months after this point, another 10 implants were inserted into both the maxilla and mandible. Two months later, all implants had once again failed to osseointegrate. During the attempt of removal by the dentist, one of the implants was displaced into the maxillary sinus, without the dentist being able to retrieve it. The patient then visited another dentist, who could not either remove the migrated implant.

**Fig. 1:**
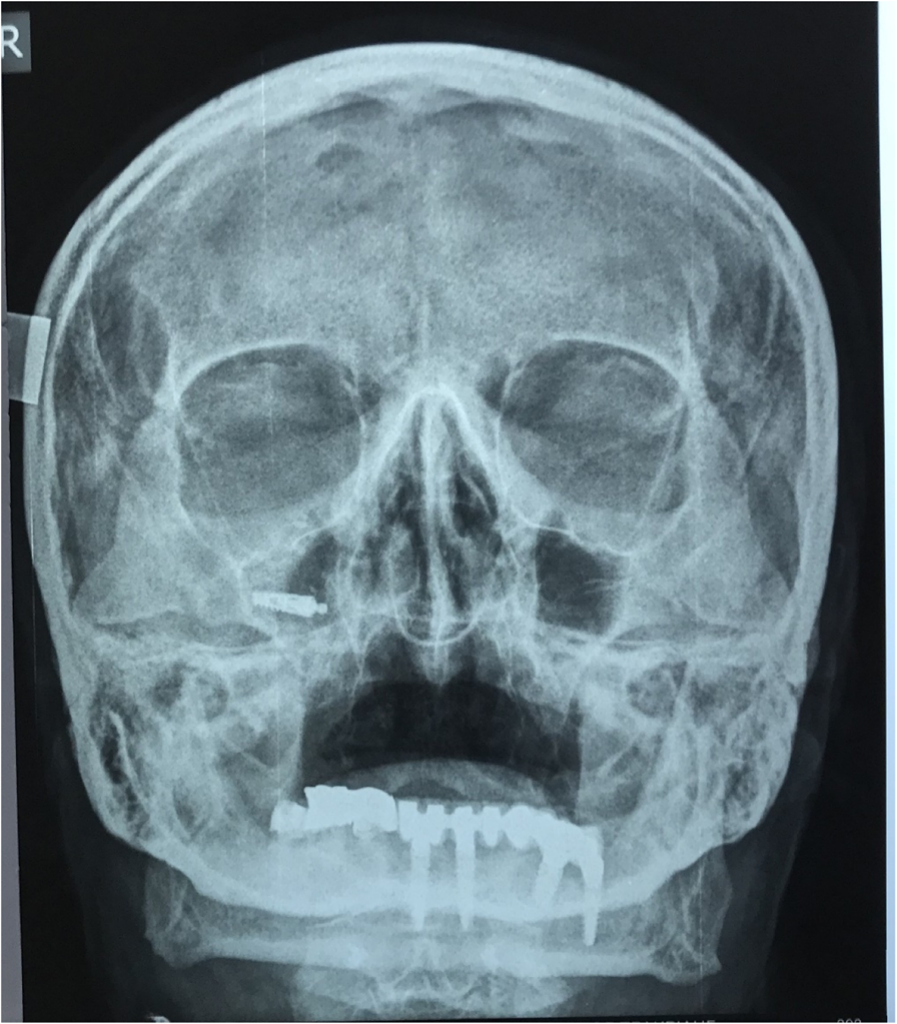
Water’s X-ray which confirmed the migrated implant into the right maxillary sinus

**Fig. 2:**
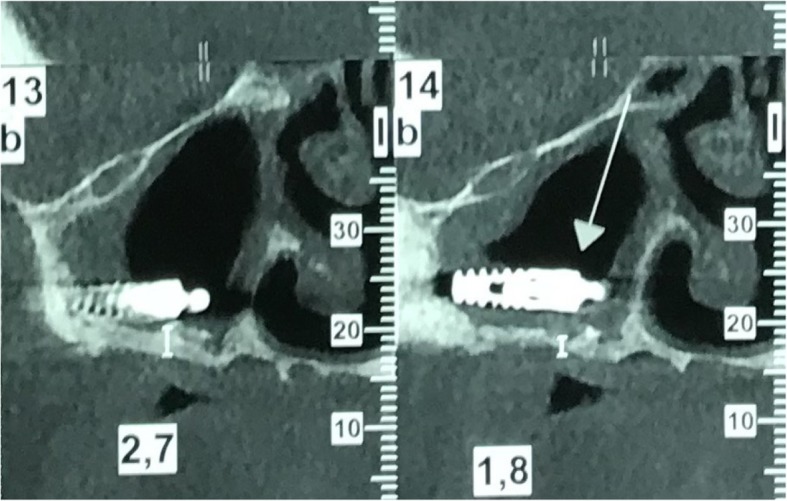
The exact position of the displaced implant into the maxillary sinus was made known after performing CBCT

Upon arrival to our clinic, a full medical and dental record was retrieved from the patient, and he was scheduled for surgical removal of the implant. Prior to operation, the patient underwent a radiographic examination with water’s X-ray (Fig. 1), as well as CBCT examination (Fig. 2) which confirmed implant migration and revealed its exact position inside the maxillary sinus.

**Fig. 3:**
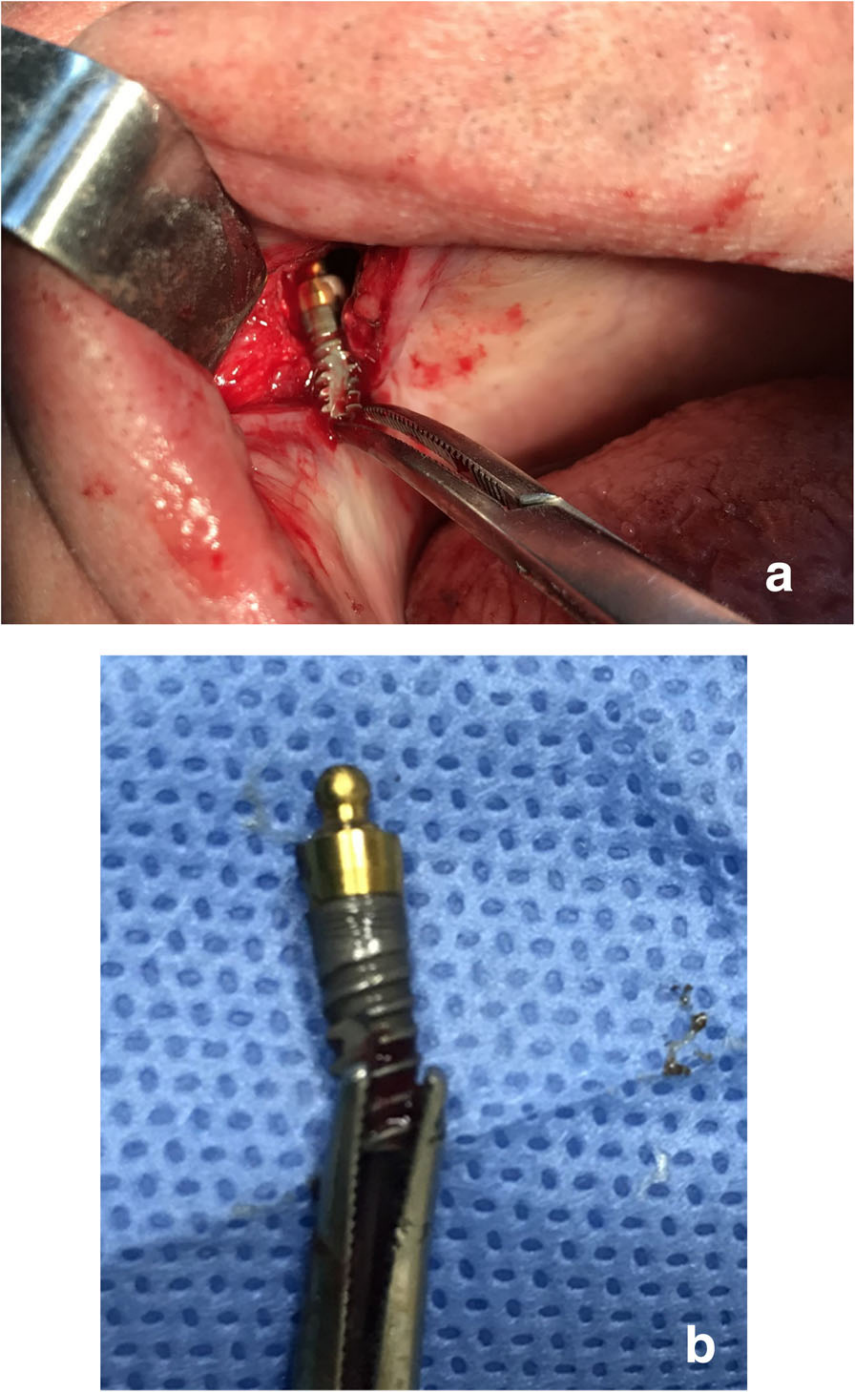
**a** Capture of the migrated implant through the bony window. **b** The implant after it was removed from the right maxillary sinus

**Fig. 4:**
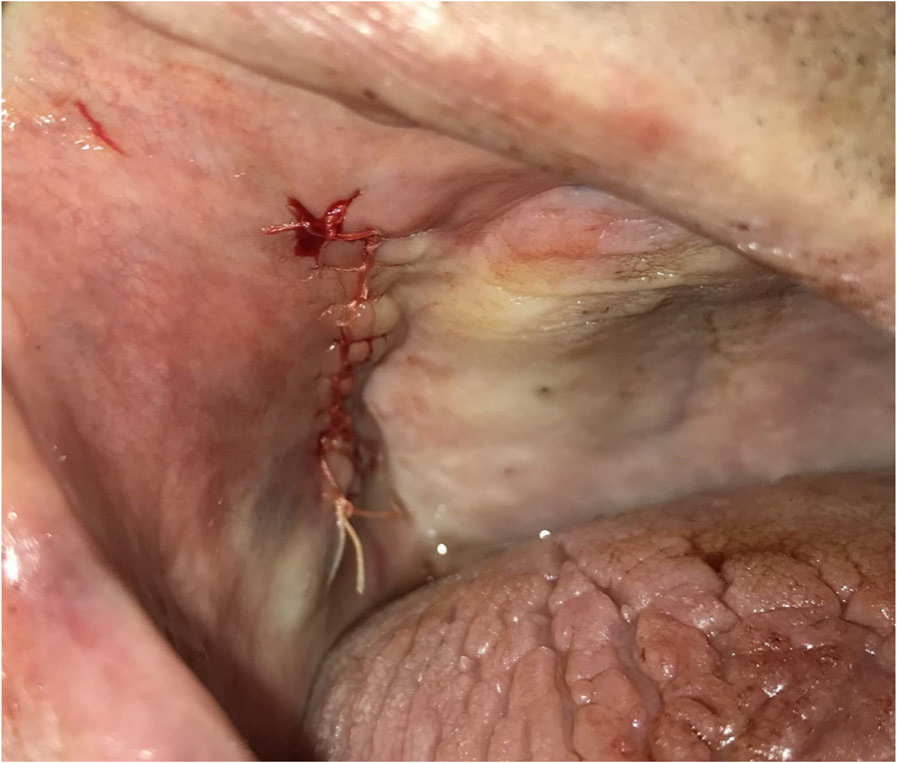
Surgical site after primary closure with resorbable sutures

Surgical procedure initiated with local anesthesia by injecting xylocaine 1% andepinephrine 1:100,000 solution in the soft tissues involving the right half of the maxilla. After a crestal incision, a full-thickness mucoperiosteal flap was raised, exposing the anterior-lateral wall of the maxilla in an area extending from canine to molar region. Using a high-speed rotary instrument under sterile saline solution irrigation, a rectangular window was created in the anterior-lateral maxillary wall. The implant was detected through the bony window and captured by a mosquito forceps (Fig. 3). The mucoperiosteal flap was then placed back at its initial position and was anchored with 4.0 resorbable sutures (Fig. 4). Amoxicillin (1 g twice daily) was prescribed for 1 week with analgesic treatment. Sutures were removed 2 weeks after surgery. The patient was advised to follow a soft diet plan for 4 weeks and was provided with proper oral hygiene instructions. He underwent scheduled visits on a monthly basis to check the course of healing for the following 6 months.

### Case 2

**Fig. 5:**
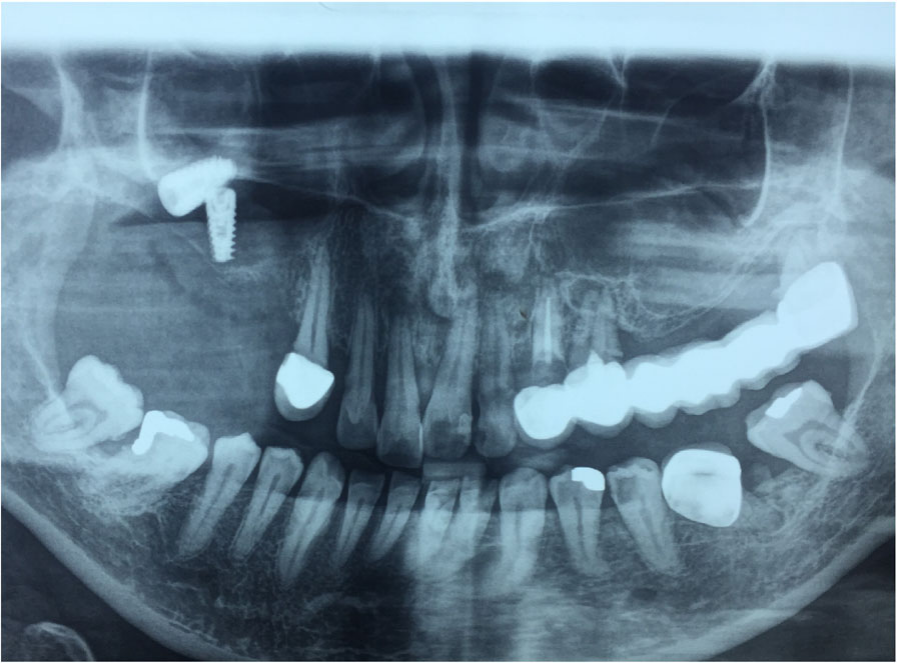
Detection of the two implants inside the right maxillary sinus

**Fig. 6:**
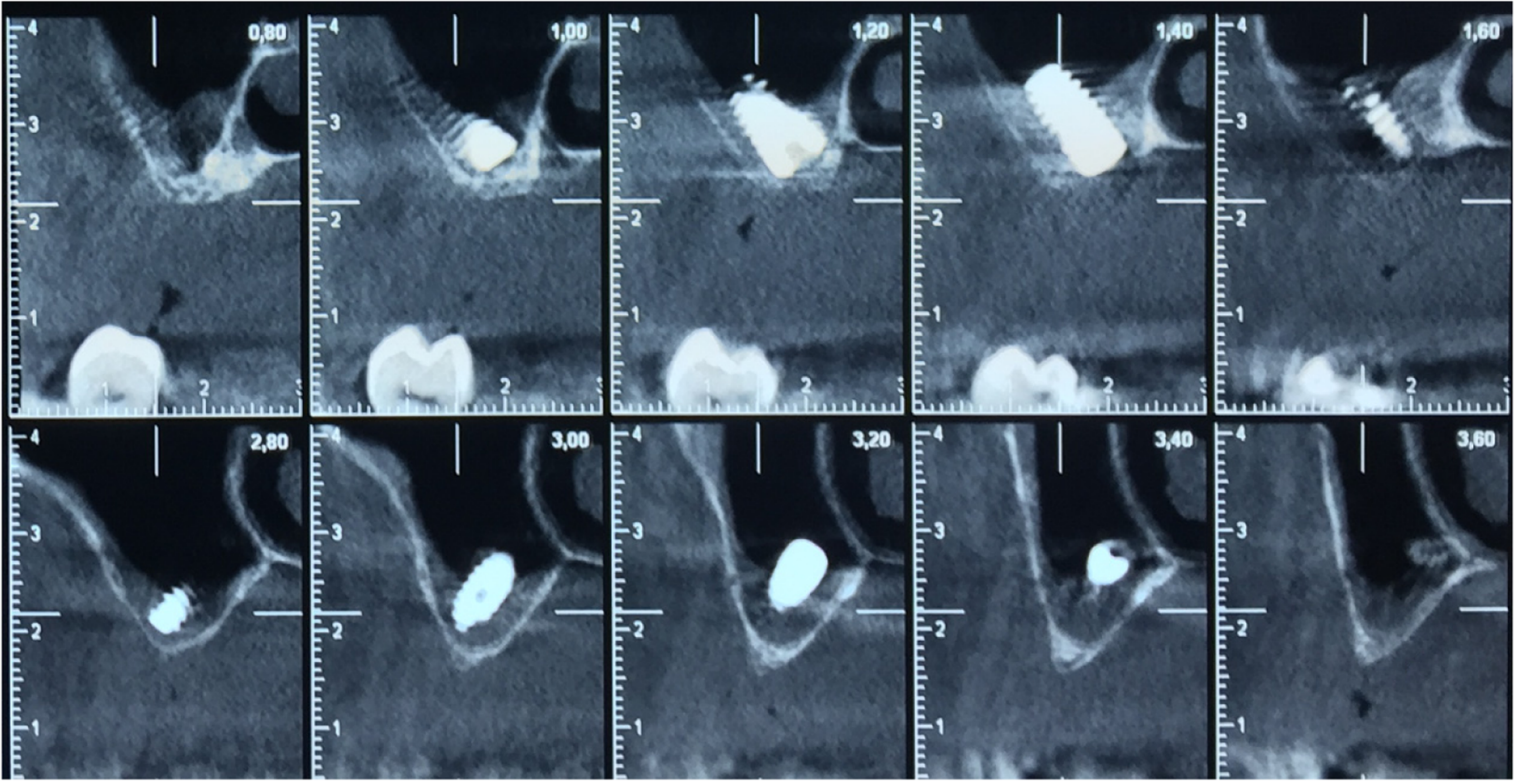
Revelation of the exact position of both implants inside the right maxillary sinus with a CBCT

A 55-year-old female patient underwent a radiographic examination with a panoramic X-ray prior to the exposure of two bone level implants which were placed by a general dentist, when their migration into the right maxillary sinus was revealed (Fig. 5). Dental scan confirmed the full migration of both implants into the maxillary sinus around the areas of #15 and #17 (Fig. 6). The implants were mobile inside the sinus, and although the patient was asymptomatic, there were signs of mucosal thickening in the CBCT.

**Fig. 7:**
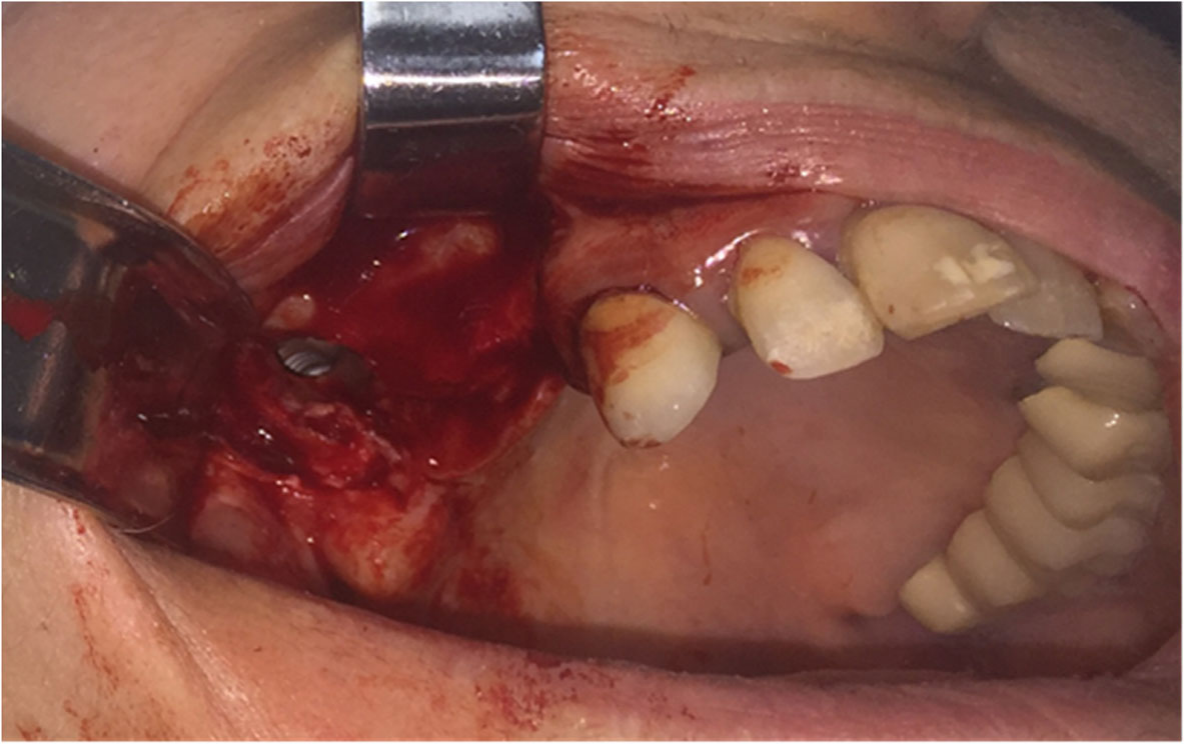
Exposure of implants after osteotomy of the maxillary sinus and removal of the sinus membrane

**Fig. 8:**
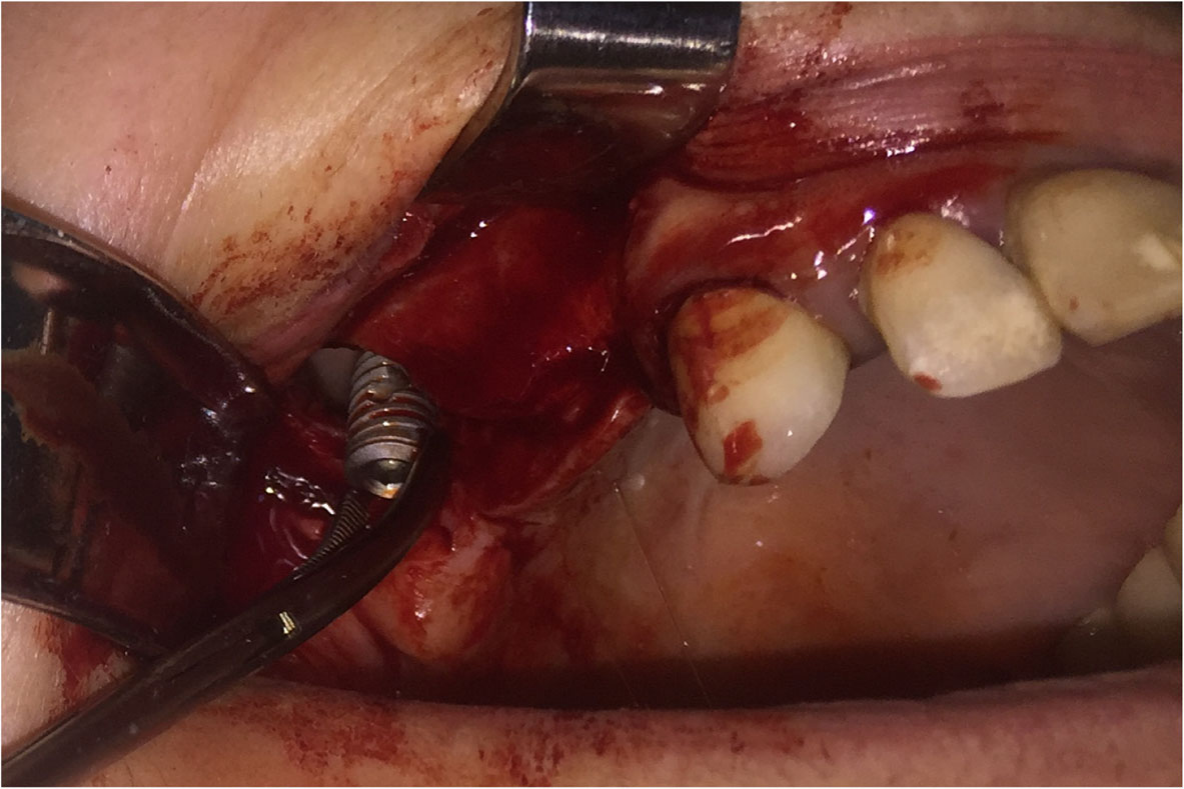
The first implant was captured and removed

**Fig. 9:**
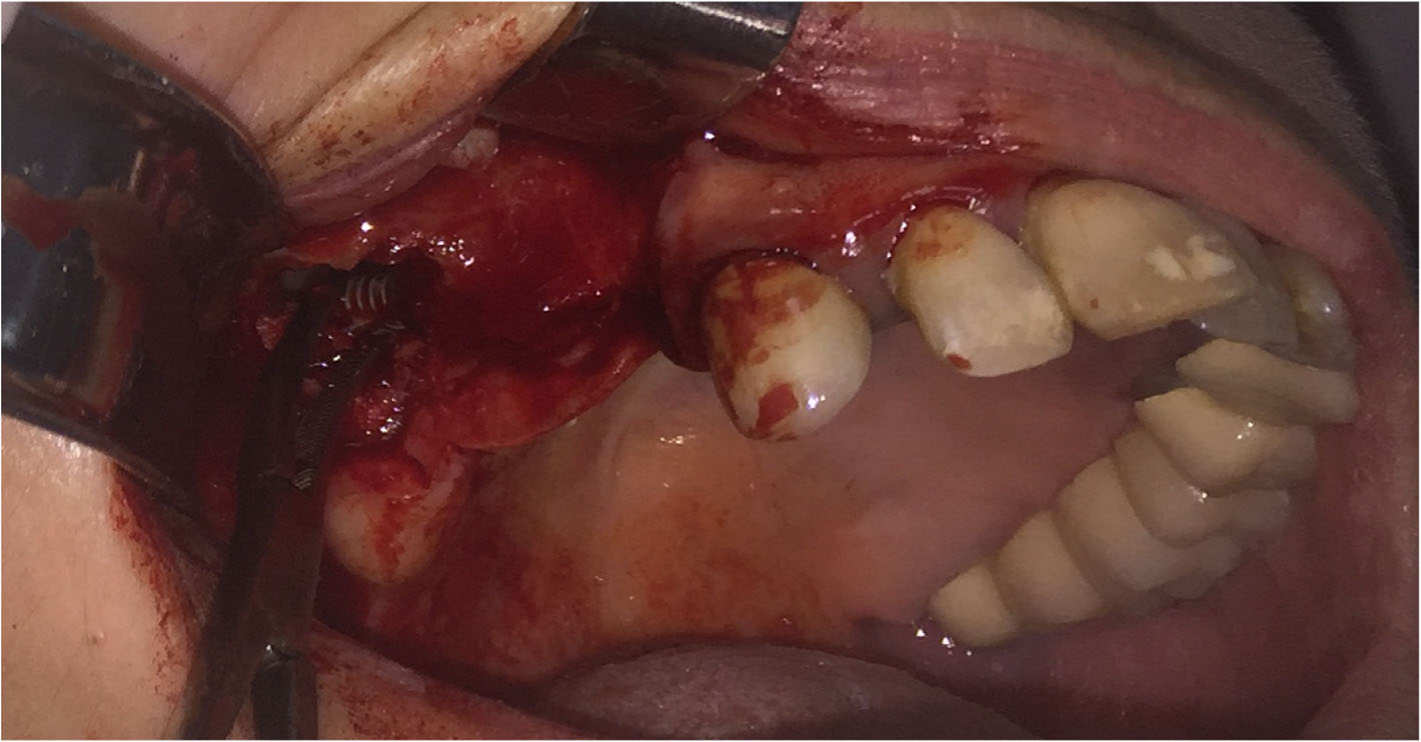
The second implant was captured and removed

**Fig. 10:**
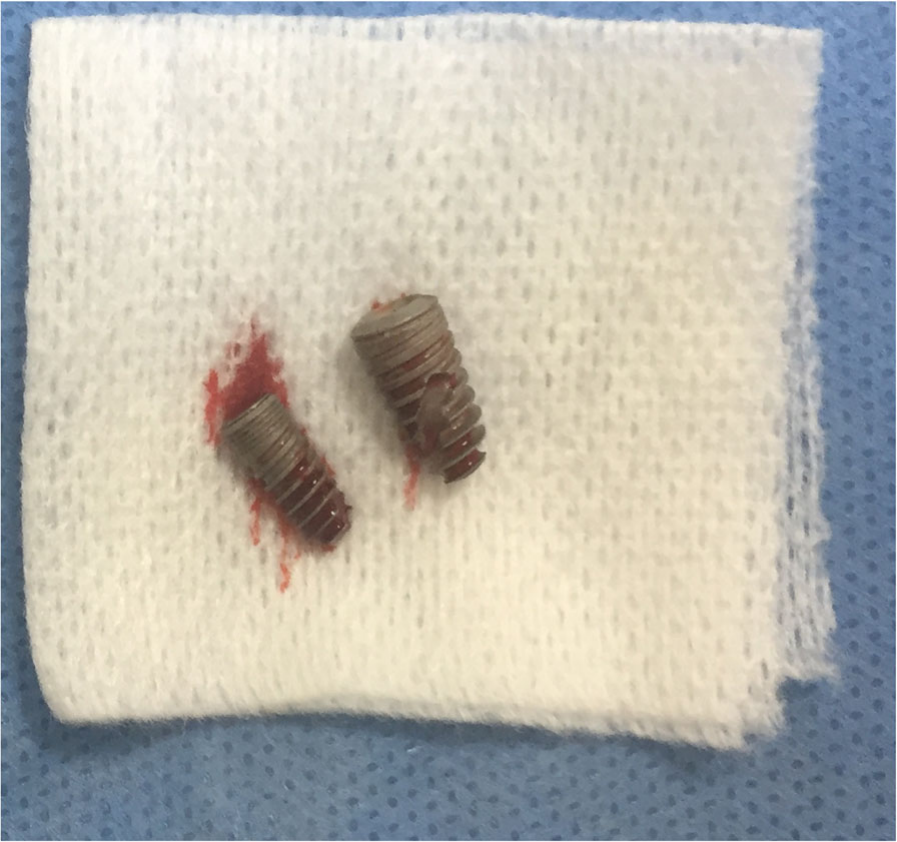
The removed implants

After the administration of a local anesthetic solution (2% lidocaine with 1:100,000 epinephrine), a vestibular incision was made, and a mucoperiosteal flap was raised to expose the lateral bony wall of the right maxillary sinus. Osteotomyperpendicularly to this wall was then performed. The bony window was detached carefully and after removal of the sinus membrane, both implants were exposed (Fig. 7). The implants were then captured with a mosquito forceps (Figs. 8 and 9). Both implants were successfully removed from the right maxillary sinus (Fig. 10). The mucoperiosteal flap was anchored to its initial position with 4.0 resorbable sutures to achieve passive primary wound closure. Amoxicillin (1 g twice daily) was prescribed for 1 week with analgesic treatment. Sutures were removed 10 days postoperatively. Visits on a monthly basis were then scheduled to check the course of healing for 6 months.

## Discussion

The incidence of implant displacement into the maxillary sinus remains unknown because of the lack of cohort studies and the relatively few published case reports [9]. However, the fact that the number of publications on this subject over the past 8 years has doubled compared to previous years, might imply a tendency toward an increasing incidence of implant displacement inside the maxillary sinus. An obvious explanation for this finding could be the rise in the number of patients treated with dental implants over the last years in combination with the fact that implant placement is performed by dentists with short of experience in many cases [11].

Implant displacement into the maxillary sinus can occur intraoperatively or postoperatively either prior to implant loading of after functional loading [9, 10].

Several factors can lead to this complication. At the intraoperative stage implant migration to the maxillary sinus can be a result of incorrect surgical planning (placement of implants in sites with inadequate bone height and volume), surgical inexperience of the anatomic landmarks of the maxillary sinus and improper surgical procedures (overpreparation of the recipient site, application of heavy force during implant placement, perforation of the sinus membrane during drilling sequence) [9]. Moreover, an unsuccessful sinus floor elevation procedure can result in uneven bone regeneration leading to less residual bone for implantation [8, 16]. These factors can affect primary stability of the implant, which is the main cause of implant migration at this stage by allowing implant micromovement that prevents clot formation and revascularization and thus inhibits new bone formation [9, 17]. Primary stability is defined as the absence of mobility in the bone bed after the implant has been placed [18]. It depends on the mechanical engagement of an implant with the fresh bone socket [19]. Besides the adequate level of surgical experience and the way the surgical procedure is performed, primary stability is affected also by the quantity and quality of the bone [18, 20], implant morphology [18], implant surface roughness, and topography [21, 22]. As a result, lack of primary stability can lead to early failure of the implant which in combination with the close proximity of the implant with the maxillary sinus may eventually lead to implant migration into the sinus. Lack of primary implant stability may be the cause of osseointegration failure in most cases, as well as in both cases that are demonstrated in the present paper. This notion can be derived by examining the bone height in CBCTs of both patients. It seems also that sinus floor elevation in case 1 was unsuccessful since there are no signs of bone regeneration into the right sinus.

At the postoperative stage, prior to implant loading, migration can be caused by impaired osseointegration as a result of infection, clinical, or subclinical oroantral fistulae and sinusitis [9]. Moreover, failure in osseointegration can be the result of pre-existent infection of the bone at the site of implant placement leading to bone destruction, or a particular deterioration of the bone structure such as osteoporosis or osteopenia [2]. Last but not least, there is one report of implant migration at the time of abutment connection due to the lack of implant osseointegration [23].

Migration of an implant after functional loading is extremely rare, and it is usually related to implant fracture [10], incorrect masticatory forces, exert destructive forces on the bone around the implant and implant loading in a time interval of less than 3 weeks after placement [2, 23].

Concerning implantation planning, the most frequently involved site associated with implant migration into the maxillary sinus is the upper first molar area (58.3%), followed by second premolar, second molar (16.6%), and first premolar (8.3%) sites [9]. The most frequently displaced implants are shown to be cylindrical implants (62.5%) without the association of the implant diameter with the fact of displacement [9]. Regarding implant length, most displaced implants are shown to be more than 10 mm in length size, while shorter implants appear to have a smaller incidence of implant migration [9]. Therefore, caution is needed especially in the posterior maxilla where many authors suggest the use of wider and longer implants due to the poor bone quality in this area (IV bone type) [23, 24]. Taking bone quantity into account, the height of residual bone is a key factor when deciding about implant length [9]. In any case, the inserted implant must not penetrate more than 4 mm into the maxillary sinus in order to prevent sinusitis or implant migration [15].

In the aim to explain the migration of an implant into the maxillary sinus, three main mechanisms have been proposed [2]. One is the changes in the intrasinus and nasal pressure: such changes can produce a suction effect because of the negative pressure exerted by the intrasinus and nasal cavity [2, 25]. Another mechanism is based on the autoimmune reaction to the implant, taking into account bone destruction secondary to infections at the implant site either before or after the implantation [2]. Such an example is the pre-existent apical foci involving the teeth, producing osteitis and bone weakening with resorption of certain parts of the maxilla, or peri-implantitis that leads to progressive resorption of the bone around the implant, eventually compromising osseointegration [2, 23]. One last mechanism may be the incorrect distribution of occlusal forces produced by the prosthetic restoration [26].

Since implants may move inside the sinus due to postural reasons, a CBCT should be performed immediately before surgical management, thus facilitating the location of the implants intraoperatively. Concerning the two cases that are discussed in the present paper, this was not possible since CBCT was not available in the hospital. Therefore, it was performed within 48 h prior to surgery.

Concerning the time of surgical management, one should take into account that foreign bodies in the paranasal sinuses, such as implants, should be removed immediately because, although they may remain asymptomatic for a long period, they may also lead to several complications, the most common of which is sinusitis [1–3, 7–13, 23]. Sinusitis is caused due to interruption of the mucociliary clearance or due to tissue reaction [7, 9]. It can also facilitate bacterial colonization, or even fungal infections, such as aspergillosis [7, 9, 27, 28]. Maxillary sinus infection can further lead to orbital cellulitis and damage of the optic nerve [29]. There has also been reported one case of cluster-like-headache associated with implant migration in the maxillary sinus [30]. A study on the tissues from the removed implant threads has reported various degenerative changes in the maxillary sinus mucosa associated with chronic inflammation, while others state that a foreign body inside the maxillary sinus can lead to cancer because of chronic irritation [7, 9, 31, 32].

Management of implant displacement into the maxillary sinus can be achieved with the implementation of four different surgical techniques, as follows. The first one involves functional endoscopic sinus surgery [9, 11, 12]. This procedure begins with a partial uncinectomy and middle meatal antrostomy with enlargement of the maxillary sinus ostium. By these means, better access to the maxillary sinus is achieved and the displaced implant can be retrieved [11]. This procedure is suitable for cases with implant displacement into the maxillary sinus without the existence of oroantral communication [9, 11]. The choice for performing endoscopic implant retrieval is not affected by symptoms of paranasal sinusitis and/or obstruction of the natural maxillary ostium [11]. Advantages of this procedure are the following: (a) the fact that it is a less invasive procedure, (b) the possibility of endoscopic control and treatment of maxillary antrum, nasal mucosa, ethmoid cells, frontal sinus, and sphenoidal sinus pathology, (c) the surgical ‘toilette’ and enlargement of the obstructed maxillary ostium, and (d) the quick recovery of maxillary sinus functions [11]. However, this procedure might lead to some complications, including the formation of ‘synechias’ due to scar formation between the inferior turbinate and the nasal septum [9, 11].

The second procedure is based on the intraoral removal of the displaced implant by performing the Caldwell-Luc technique [11]. This involves the creation of a bony window in the anterior-lateral aspect of the maxillary sinus and the retrieval of the displaced implant through that window [11]. The main limitation of this surgical procedure is the obligatory absence of any signs and symptoms of paranasal sinusitis and the patency of the maxillary ostium [11].

In case of a pre-existent oroantral fistula, and only when a sinus pathology is absent, implant retrieval can also be managed by taking advantage of the existing communication between the maxillary sinus and the oral cavity through the alveolar bone [9].

Finally, the fourth choice of surgical management of this complication involves the combination of the endoscopic and the intraoral approach (Caldwell-Luc technique). This combined procedure is being performed whenever implant displacement into the maxillary sinus is associated with signs and symptoms of sinusitis, obstruction of the maxillary ostium and oroantral communication [11].

## Conclusion

The displacement of an implant into the maxillary sinus occurs unexpectedly, and it is rather difficult to treat. Although such a complication used to be rare and sporadic, its incidence has increased drastically over the past decade. Therefore, it is important to accurately evaluate the specific characteristics of the patient and the bone site prior to implantation planning. Under this notion, the surgeon must assess possible difficulties that may appear intraoperatively and refer the patient to a more experienced doctor if necessary. Once this incidence occurs, the migrated implant must be removed from the maxillary sinus since it can be eventually the cause of late sinusitis due to foreign body reaction which can take place many years later [10, 33].
